# Propagation of terahertz waves in a monoclinic crystal BaGa_4_Se_7_

**DOI:** 10.1038/s41598-018-34552-y

**Published:** 2018-11-01

**Authors:** Yiwen E, Jiyong Yao, Li Wang

**Affiliations:** 1 Beijing National Laboratory for Condensed Matter Physics, Institute of Physics, Chinese Academy of Sciences, Beijing, 100190 China; 2 Key Laboratory of Functional Crystals and Laser Technology, Technical Institute of Physics and Chemistry, Chinese Academy of Sciences, Beijing, 100190 China; 3 School of Physical Sciences, University of Chinese Academy of Sciences, Beijing, 100049 China

## Abstract

The complex symmetric dielectric tensor of a monoclinic crystal cannot be diagonalized by a space rotation operation in general, which poses a serious difficulty in analyzing the propagation of electromagnetic fields in monoclinic crystals so far. This propagation issue is discussed in a general case without using the index ellipsoid scheme. It is found that, when incident waves travel along the mirror plane normal or 2-fold rotation axis of monoclinic crystals, two eigenmodes following specific dispersion relations are elliptically polarized with the same ellipticity and chirality but have spatially orthogonal elliptical principal axes. The frequency independent features are the unique manifestation of the crystal symmetry. Using polarization sensitive terahertz time-domain spectroscopy and our developed data analyzing and processing methods, three complex permittivity tensor elements for a monoclinic crystal BaGa_4_Se_7_ are straightforwardly extracted and the properties of the two eigenmodes are characterized in full. It is also interesting that the spectral components beyond 1.7 THz show a very high refraction index (>10) and low dissipation during propagation, which suggests that the bulk phonon-polariton waves may be excited and effectively propagate in the crystal, resulting from the coherent phonon excitations by the incident terahertz waves. Our results may promote to develop novel terahertz devices based on polariton excitation and propagation in monoclinic crystals.

## Introduction

Electromagnetic (EM) field propagating in a medium carries fundamental information about the interaction with matter. During propagation, all the properties, such as absorption, dispersion and polarization states, are determined by the complex permittivity (for non-magnetic materials) in constitutive relations associated with Maxwell’s equations^1^. In practice, the frequency domain quantity *ε*(*ω*) is often expressed in terms of a complex refractive index, $\varepsilon (\omega )\equiv {[\tilde{n}(\omega )]}^{2}={[n(\omega )-i\kappa (\omega )]}^{2}$, where *n*(*ω*) is the refractive index and *κ*(*ω*) is the extinction coefficient describing the dispersion and absorption of the media, respectively. In isotropic media or cubic crystals, a scalar dielectric parameter *ε*(*ω*) can fully describe the EM field propagation properties, and the polarization state of an arbitrary traveling field keeps unchanged. The situation becomes complicated when anisotropic materials are considered, where *ε*(*ω*) is a complex second-order tensor and, in principle, the optical parameters *n*(*ω*) and *κ*(*ω*) can only be defined for specific propagating modes, i.e., eigenmodes^2^. An eigenmode is a special solution of Maxwell’s equations and satisfies a specific dispersion relation characterized by *ε*(*ω*), and has well-defined absorption and dispersion properties, and keeps its polarization state constant during propagation. For many birefringence crystals, which are classified as uniaxial or biaxial crystals, the dielectric tensor *ε*(*ω*) can be diagonalized by space rotation transforms to establish the three principal axes^3^. In this situation, the eigenmodes are linearly polarized fields with the polarization along each principal axis. Any propagating field can be decomposed into a superposition of two independent eigenmodes with orthogonal polarizations. This treatment is useful and even necessary to facilitate the analysis of propagation process. Another example is the EM waves propagating in an isotropic chiral medium, where an extra magnetoelectric coupling coefficient is introduced into constitutive relations to describe the cross-coupling between electric and magnetic fields^4^. In this case, two different handed circular-polarized waves serve as eigenmodes with different dispersion relations.

In monoclinic crystals, there are two crystallographic axes that are not orthogonal to each other, and the dielectric tensor contains off-diagonal elements when expressed in the dielectric frame. Because the dielectric tensor is a complex symmetric matrix in general, it cannot be diagonalized by a space rotation operation. This situation poses a serious difficulty in analyzing the propagation of EM fields in monoclinic crystals^5,6^. It is troublesome to obtain the tensor elements from experiment measurements^7–9^. If absorption is low enough to be negligible, the dielectric tensor *ε*(*ω*) is considered as a real symmetric one and can be diagonalized^10,11^. In this case, the orientation of principal axis system is frequency dispersive. The problem related to monoclinic crystals is mainly originated from the difficulty to obtain sufficient information in optical measurements. In terahertz (THz) frequency region, the absorption in many materials cannot be ignored and the complex dielectric tensor *ε*(*ω*) has to be dealt with. But using terahertz time-domain spectroscopy (THz-TDS), both the amplitude and the phase of THz EM fields can be directly measured in experiments. Comparing to optical measurements where the phase information cannot be measured in most cases, the collected information by THz-TDS measurements is doubled. It is a huge advantage in studying the EM responses of monoclinic crystals.

In the paper, we study the properties of a THz EM field propagating along the symmetric axis of monoclinic crystals. The dispersion relations for two eigenmodes are derived from the Maxwell’s equations. The structures and properties of eigenmodes are analyzed and discussed. The monoclinic crystal BaGa_4_Se_7_ (BGSe) is taken as an example for THz-TDS polarization measurements. Data acquisition and analysis procedure are presented in detail. The three tensor elements of the dielectric tensor *ε*(*ω*), as well as the complex refractive index for both eigenmodes, are extracted from the experimental data. The probable excitation and propagation of phonon-polaritons are also discussed.

## Results and Discussion

### Eigenmodes in monoclinic crystals

**Figure 1:**
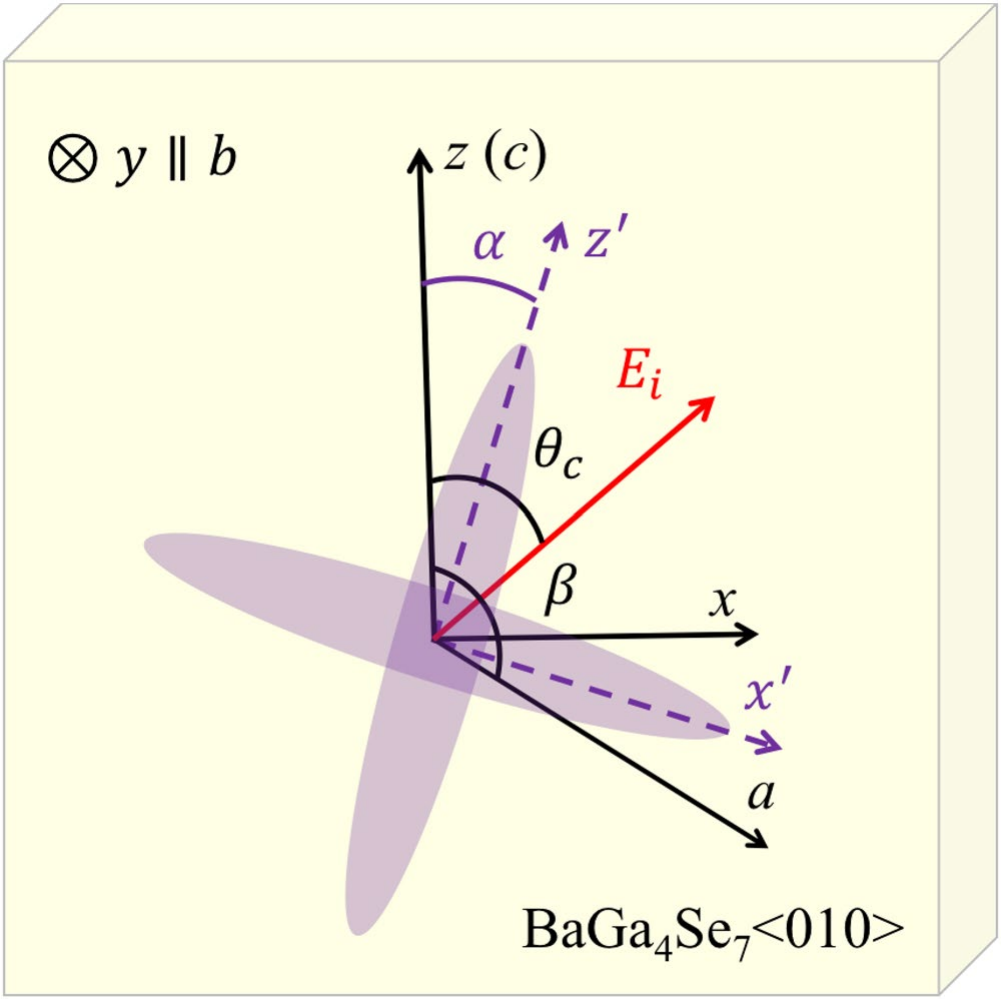
Crystallographic (*a*, *b*, *c*) and dielectric frames (*x*, *y*, *z*) of the crystal. The crystallographic axis *b* is perpendicular to the ac-plane and the angle *β* between *a* and *c* axis is 121.24°. The dielectric axes *y* and *z* are parallel to the *b* and *c* axes, respectively. *E*_*i*_ shows the polarization of linearly polarized incident field. The angle *α* describes the major axis direction for one of the elliptically polarized eigenmodes.

Without loss of generality, the monoclinic crystal BGSe is used for theoretical analysis and experimental measurements. BGSe has a mirror symmetric (010) plane in the dielectric frame and its dielectric tensor is expressed as

1 $$\varepsilon (\omega )=(\begin{array}{lll}{\varepsilon }_{xx} & 0 & {\varepsilon }_{xz}\\ 0 & {\varepsilon }_{yy} & 0\\ {\varepsilon }_{zx} & 0 & {\varepsilon }_{zz}\end{array}),$$

where *ε*_*xz*_ = *ε*_*zx*_, *y* and *z* axis correspond *b* and *c* axis in crystallographic frame, but the *x* axis does not align with the *a* axis as shown in Fig. 1.

Suppose a plane EM wave is traveling along *y* axis in the crystal, ${\boldsymbol{E}}(y)=({E}_{x}{\boldsymbol{x}}+{E}_{z}{\boldsymbol{z}}){e}^{i{k}_{y}y}$, where *E*_*x*_ and *E*_*z*_ are complex component projections in *x* and *z* axis, ***x*** and ***z*** are unit vectors in the dielectric frame, respectively. From Maxwell’s equations and the constitutive relation, we have

2 $$(\begin{array}{ll}{\omega }^{2}{\varepsilon }_{0}{\mu }_{0}{\varepsilon }_{xx}-{k}_{y}^{2} & {\omega }^{2}{\varepsilon }_{0}{\mu }_{0}{\varepsilon }_{xz}\\ {\omega }^{2}{\varepsilon }_{0}{\mu }_{0}{\varepsilon }_{xz} & {\omega }^{2}{\varepsilon }_{0}{\mu }_{0}{\varepsilon }_{zz}-{k}_{y}^{2}\end{array})(\begin{array}{c}{E}_{x}\\ {E}_{z}\end{array})=0,$$

where *ε*_0_ and *μ*_0_ are vacuum permittivity and permeability. The existing nontrivial solutions of the electric field require the determination of the matrix in equation (2) must equal to zero, which results in the dispersion relations:

3 $${k}_{y}^{\pm }=\frac{\omega }{c}\sqrt{\frac{{\varepsilon }_{xx}+{\varepsilon }_{zz}\pm {\varepsilon }_{m}}{2}}\equiv {k}^{\pm }$$

with ${\varepsilon }_{m}=\sqrt{{({\varepsilon }_{xx}-{\varepsilon }_{zz})}^{2}+4{\varepsilon }_{xz}^{2}}$. It is easy to verify that ${k}^{+}=\frac{\omega }{c}\sqrt{{\varepsilon }_{xx}}$ and ${k}^{-}=\frac{\omega }{c}\sqrt{{\varepsilon }_{zz}}$ when *ε*_*xz*_ = 0. The dispersion relations shown in equation (3) define two eigenmodes of the EM field propagating in the crystal, which have different phase velocities, dissipations and polarization states and are labeled as “±” in this paper respectively. The field components *E*_*x*_ and *E*_*z*_ of the eigenmodes satisfy the following relations,

4 $$\frac{{E}_{z}^{\pm }}{{E}_{x}^{\pm }}=\frac{-({\varepsilon }_{xx}+{\varepsilon }_{zz}\pm {\varepsilon }_{m})}{2{\varepsilon }_{xz}}\equiv {\sigma }_{\pm }.$$

The field amplitude ratios *σ*_±_ are complex numbers and meet the relation *σ*_+_*σ*_−_ = −1, which is a pure manifestation of the spatial symmetry of monoclinic crystals, independent of frequency and specific crystals. The parameters *σ*_+_ and *σ*_−_ contain all the information about properties of the two eigenmodes.

An arbitrary propagating field in monoclinic crystals can be expressed as a superposition of the eigenmodes, ${\boldsymbol{E}}(y)=({E}_{x}^{+}{\boldsymbol{x}}+{E}_{z}^{+}{\boldsymbol{z}}){e}^{i{k}^{+}y}+({E}_{x}^{-}{\boldsymbol{x}}+{E}_{z}^{-}{\boldsymbol{z}}){e}^{i{k}^{-}y}$. After introducing the normalized eigen vector set (***e***^**+**^, ***e***^**−**^) and corresponding field amplitudes *E*^+^and *E*^−^, we can diagonalize the following matrix equation,

5 $$(\begin{array}{ll}{E}_{x}^{+} & {E}_{z}^{+}\\ {E}_{x}^{-} & {E}_{z}^{-}\end{array})(\begin{array}{c}{\boldsymbol{x}}\\ {\boldsymbol{z}}\end{array})=(\begin{array}{ll}{E}^{+} & 0\\ 0 & {E}^{-}\end{array})(\begin{array}{c}{{\boldsymbol{e}}}^{{\boldsymbol{+}}}\\ {{\boldsymbol{e}}}^{{\boldsymbol{-}}}\end{array}),$$

and express the propagating field in the new bases,

6 $$\begin{array}{c}{{\boldsymbol{e}}}^{{\boldsymbol{+}}}=({\boldsymbol{x}}+{\sigma }_{+}{\boldsymbol{z}})/\sqrt{1+{A}^{2}}\\ {{\boldsymbol{e}}}^{{\boldsymbol{-}}}=({\sigma }_{+}{\boldsymbol{x}}-{\boldsymbol{z}})/\sqrt{1+{A}^{2}}\end{array},({\sigma }_{+}\equiv A{e}^{i\varphi })$$

which are linearly independent but not orthonormal with each other in the complex space. We notice that each new base vector is orthonormal with the complex conjugate of the other. The corresponding field amplitudes of the two eigenmodes are:

7 $$\begin{array}{rcl}{E}^{+} & = & {E}_{x}^{+}{\sigma }_{-}-{E}_{z}^{+}=-\,{E}_{x}^{+}({\sigma }_{+}-{\sigma }_{-})\\ {E}^{-} & = & {E}_{x}^{-}{\sigma }_{+}-{E}_{z}^{-}={E}_{x}^{-}({\sigma }_{+}-{\sigma }_{-})\end{array}$$

The detail derivation of the above calculation is presented in the Supplementary Information. The two eigenmodes represented by the two eigenvectors in equation (6) are both elliptically polarized and have the same chirality and ellipticity, but the principal axes of ellipses are orthogonal with each other. Therefore, we only need to determine the polarization parameters, i.e., the major axis direction of ellipse and ellipticity for one mode. As shown in Fig. 1, the angle *α* between the principal axis of the ellipse and the z-axis satisfies the following equation for both modes^12^,

8 $$\tan (2{\alpha }^{+})=\,\tan (2{\alpha }^{-})=\frac{2A}{1-{A}^{2}}\,\cos \,\varphi .$$

There exists an intrinsic multiple of *π*/2 uncertainty in the solution. In practice, this problem can be solved by comparing the amplitudes of the experimentally measured field projections for both modes in the direction of *α* and *α* + *π*/2 solved from equation (8). Then the major and minor axes for each mode can be resolved and determined. According to the definition, the ellipticity *χ* = ±arctan(*b*/*a*), where *a* and *b* are the lengths of the major and minor axes and the sign describes the chirality of elliptical polarization. The ellipticity *χ* can be calculated as

9 $${\chi }^{+}={\chi }^{-}=\frac{1}{2}\arcsin (\frac{2A}{{A}^{2}+1}\,\sin \,\varphi )$$

### Polarization sensitive TDS measurements

**Figure 2:**
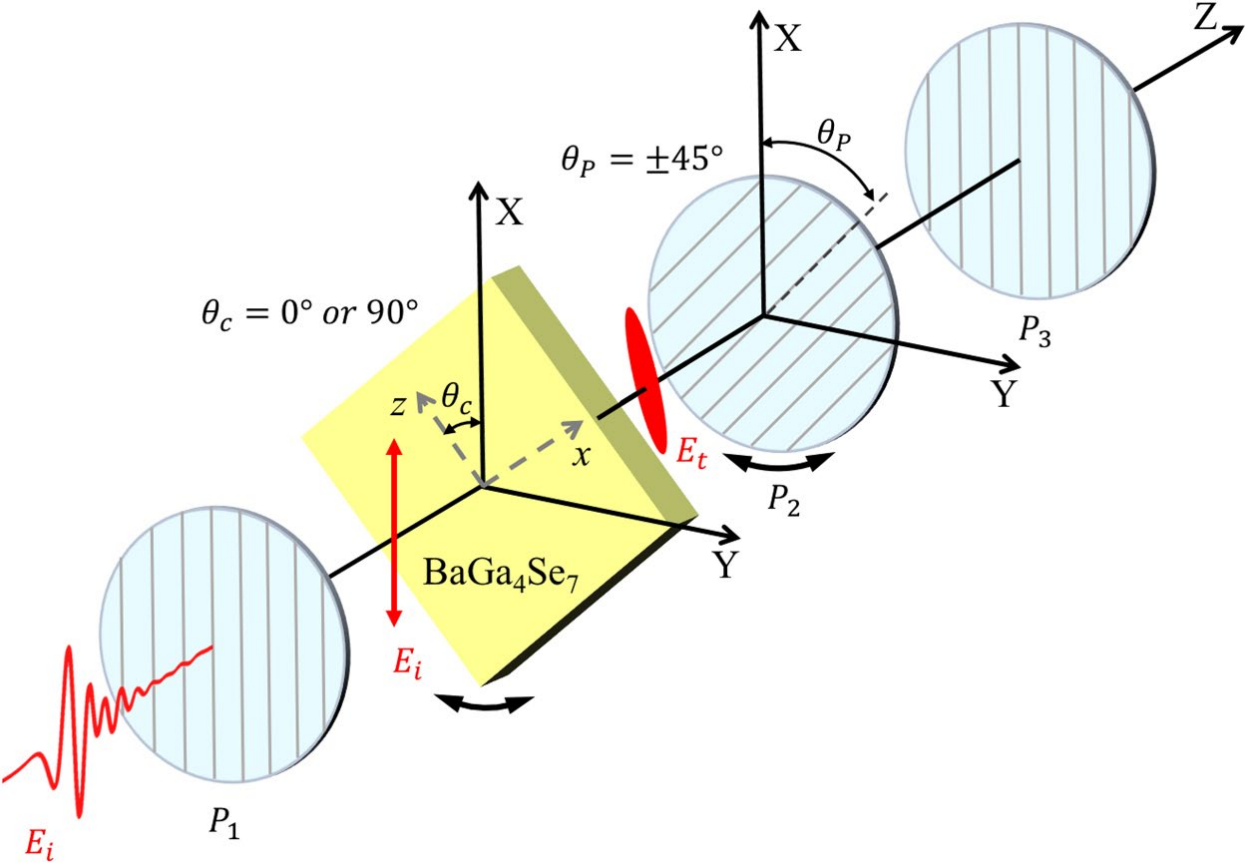
Schematic illustration of the polarization sensitive THz-TDS system. Laboratory frames are denoted by (X, Y, Z). THz polarizer *P*_1_ and *P*_3_ are fixed and have the same setting for transmitting X-polarized THz field. *P*_2_ is set to *θ*_*P*_ = ±45° to acquire two orthogonal components of the field. Note that the angle is positive (negative) if it is measured from X-axis counterclockwise (clockwise). The azimuth orientation of the crystal (BaGa_4_Se_7_) is described by the angle *θ*_*c*_.

A polarization sensitive THz-TDS system is used for the transmission measurement of BGSe (010) crystals (Fig. 2). The BGSe crystal is grown using the Bridgman-Stockbarger method without seed^13,14^. After passing the first polarizer *P*_1_, the linearly polarized THz field along the X-axis is normally incident on the BGSe (010) sample, which was mounted on a motorized rotation stage for the azimuth angle (*θ*_*c*_) dependent measurements. Then, the THz beam passes the second polarizer *P*_2_, which is used to select the THz field polarization projection component with *θ*_*P*_ = ±45°. The positive (negative) sign means the angle is measured from the X-axis in laboratory frame counterclockwise (clockwise). The third polarizer *P*_3_ has the same setting as *P*_1_, which ensures a constant measurement condition. The system was carefully calibrated for accurate orientations of the three polarizers and the sample. Especially, the z-axis in the dielectric frame of the crystal was aligned exactly coincident with the X-axis in the laboratory frame for *θ*_*c*_ = 0°. During the measurement, all the THz beam paths are enclosed in a vacuum chamber to get rid of the absorption from water vapor.

**Figure 3:**
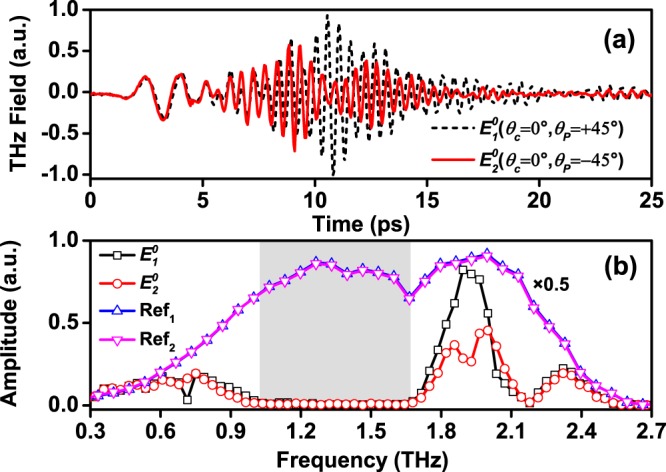
Typical THz temporal transmission waveforms (**a**) for *θ*_*P*_ is equal to ±45°, and the corresponding Fourier spectra including incident reference (Ref) counterparts (**b**). The slowly varying head part and the following fast oscillations in the temporal waveforms (**a**) correspond the separate spectral components below 1.0 THz and above 1.7 THz in (**b**). The difference of the data in the two settings is a rough measure of the polarization state deviating from a linearly polarized one.

For each sample orientation *θ*_*c*_ = 0° or 90°, two waveforms ${E}_{1}^{0}(t)$ [or ${E}_{1}^{90}(t)$] and ${E}_{2}^{0}(t)$ [or ${E}_{2}^{90}(t)$] corresponding to *θ*_*P*_ = ±45° were recorded, respectively. The reference waveform was taken by removing the sample from the beam path. The typical measured waveforms and their spectra are shown in Fig. 3 for *θ*_*c*_ = 0°. It is clear that the spectra of reference signals are unchanged for *θ*_*P*_ = ±45°, but the sample signals are significantly different. The sample waveforms have fast oscillation components with much slow propagation velocity following the low-frequency head part. The spectra are also divided into two parts accordingly. The propagation of THz waves in BGSe crystal for the spectral component below 1.0 THz and that above 1.7 THz in our data may be dominated by different mechanisms, which will be briefly discussed afterwards.

### Extraction of electromagnetic parameters

Assume a linearly polarized EM field (***E***_***i***_) is normally incident on a monoclinic crystal along *y*-axis and the angle between the polarization plane and *z*-axis is *θ*_*c*_. The Fourier amplitude of the incident electric field at the interface of y = 0 is

10 $${{\boldsymbol{E}}}_{{\boldsymbol{i}}}={E}_{0}(\sin \,{\theta }_{c}{\boldsymbol{x}}+\,\cos \,{\theta }_{c}{\boldsymbol{z}}),$$

where *E*_0_ is the amplitude of the reference THz field and can be measured directly without the sample. From equation (6), the base vectors of the dielectric frame can be expressed by the base vectors of two eigenmodes, and the incident field is decomposed into the superposition of the eigenmodes:

11 $${{\boldsymbol{E}}}_{{\boldsymbol{i}}}={E}^{+}{{\boldsymbol{e}}}^{{\boldsymbol{+}}}+{E}^{-}{{\boldsymbol{e}}}^{{\boldsymbol{-}}},$$

where

12 $$\begin{array}{rcl}{E}^{+} & = & \frac{{E}_{0}\sqrt{1+{A}^{2}}}{1+{\sigma }_{+}^{2}}({\sigma }_{+}\,\cos \,{\theta }_{c}+\,\sin \,{\theta }_{c})\\ {E}^{-} & = & \frac{{E}_{0}\sqrt{1+{A}^{2}}}{1+{\sigma }_{+}^{2}}({\sigma }_{+}\,\sin \,{\theta }_{c}-\,\cos \,{\theta }_{c})\end{array}.$$

For each eigenmode, the transmission coefficients at the two interfaces and the propagation factor are well defined. The amplitude of the transmitted electric field can be expressed as follows,

13 $${{\boldsymbol{E}}}_{{\boldsymbol{t}}}={t}_{+}{p}_{+}{t^{\prime} }_{+}{E}^{+}{{\boldsymbol{e}}}^{{\boldsymbol{+}}}+{t}_{-}{p}_{-}{t^{\prime} }_{-}{E}^{-}{{\boldsymbol{e}}}^{{\boldsymbol{-}}},$$

where *t*_+_, *t*_−_ and ${t^{\prime} }_{+}$, ${t^{\prime} }_{-}$ are the complex transmission amplitudes for each mode at the front and rear interfaces, respectively. *p*_+_ and *p*_−_ are the propagation factors. We have

14 $${t}_{\pm }{t^{\prime} }_{\pm }=\frac{4{n}^{\pm }}{{({n}^{\pm }+\mathrm{1)}}^{2}},$$

15 $${p}_{\pm }=\exp (\frac{i\omega {n}^{\pm }d}{c}),$$

where d is the thickness of the crystal sample. Substituting equations (6), (12), (14) and (15) into equation (13), we have

16 $$\begin{array}{rcl}{{\boldsymbol{E}}}_{{\boldsymbol{t}}} & = & \frac{{E}_{0}}{1+{\sigma }_{+}^{2}}[{\sigma }_{+}({T}_{+}-{T}_{-})\cos \,{\theta }_{c}+({T}_{+}+{\sigma }_{+}^{2}{T}_{-})\sin \,{\theta }_{c}]{\boldsymbol{x}}\\  &  & +\frac{{E}_{0}}{1+{\sigma }_{+}^{2}}[{\sigma }_{+}({T}_{+}-{T}_{-})\sin \,{\theta }_{c}+({\sigma }_{+}^{2}{T}_{+}+{T}_{-})\cos \,{\theta }_{c}]{\boldsymbol{z}},\end{array}$$

where ${T}_{\pm }\equiv {t}_{\pm }{t}_{\pm }^{^{\prime} }{p}_{\pm }$ are the transmission functions of eigenmodes. In equation (16), the quantities *E*_*t*_ and *E*_0_ are directly measured and *θ*_*c*_ can be set freely. The unknown quantities are *σ*_+_, *T*_+_ and *T*_−_, all of which are functions of *ε*_*xx*_, *ε*_*zz*_, and *ε*_*xz*_. Therefore, once three independent measurements of *E*_*t*_ are performed, *σ*_+_, *T*_+_ and *T*_−_, can be worked out. And the three matrix elements of tensor *ε*(*ω*) are determined subsequently by using equations (3), (4), (14) and (15).

By setting the azimuth angle of the sample to *θ*_*c*_ = 0° and 90° (see Fig. 2), the two projection components of transmitted fields from the sample for *θ*_*P*_ = ±45°, denoted by *E*_1_ and *E*_2_, were measured. From equation (16), the following equations were built,

17 $${E}_{tz}^{0}=\frac{{E}_{0}}{1+{\sigma }_{+}^{2}}({\sigma }_{+}^{2}{T}_{+}+{T}_{-})=\frac{\sqrt{2}}{2}({E}_{1}^{0}+{E}_{2}^{0}),$$

18 $${E}_{tx}^{0}=\frac{{\sigma }_{+}{E}_{0}}{1+{\sigma }_{+}^{2}}({T}_{+}-{T}_{-})=\frac{\sqrt{2}}{2}({E}_{2}^{0}-{E}_{1}^{0}),$$

19 $${E}_{tx}^{90}=\frac{{E}_{0}}{1+{\sigma }_{+}^{2}}({T}_{+}+{\sigma }_{+}^{2}{T}_{-})=\frac{\sqrt{2}}{2}({E}_{1}^{90}+{E}_{2}^{90}),$$

20 $${E}_{tz}^{90}=\frac{{\sigma }_{+}{E}_{0}}{1+{\sigma }_{+}^{2}}({T}_{+}-{T}_{-})=\frac{\sqrt{2}}{2}({E}_{1}^{90}-{E}_{2}^{90}),$$

where all the field components are frequency domain quantities and the superscripts stand for the values of *θ*_*c*_. The subscript *x* and *z* indicate the projection components on the corresponding axis in dielectric frame of the crystal. It is noticeable that ${E}_{tx}^{0}$ equals to ${E}_{tz}^{90}$ as shown in equations (18) and (20). We checked the experiment data of the right side of the equations and confirmed that they were identical, which is a proof of reliability and robustness of our measurement system. The three independent equations (17)–(19) are sufficient to calculate *σ*_+_, *T*_+_ and *T*_−_.

**Figure 4:**
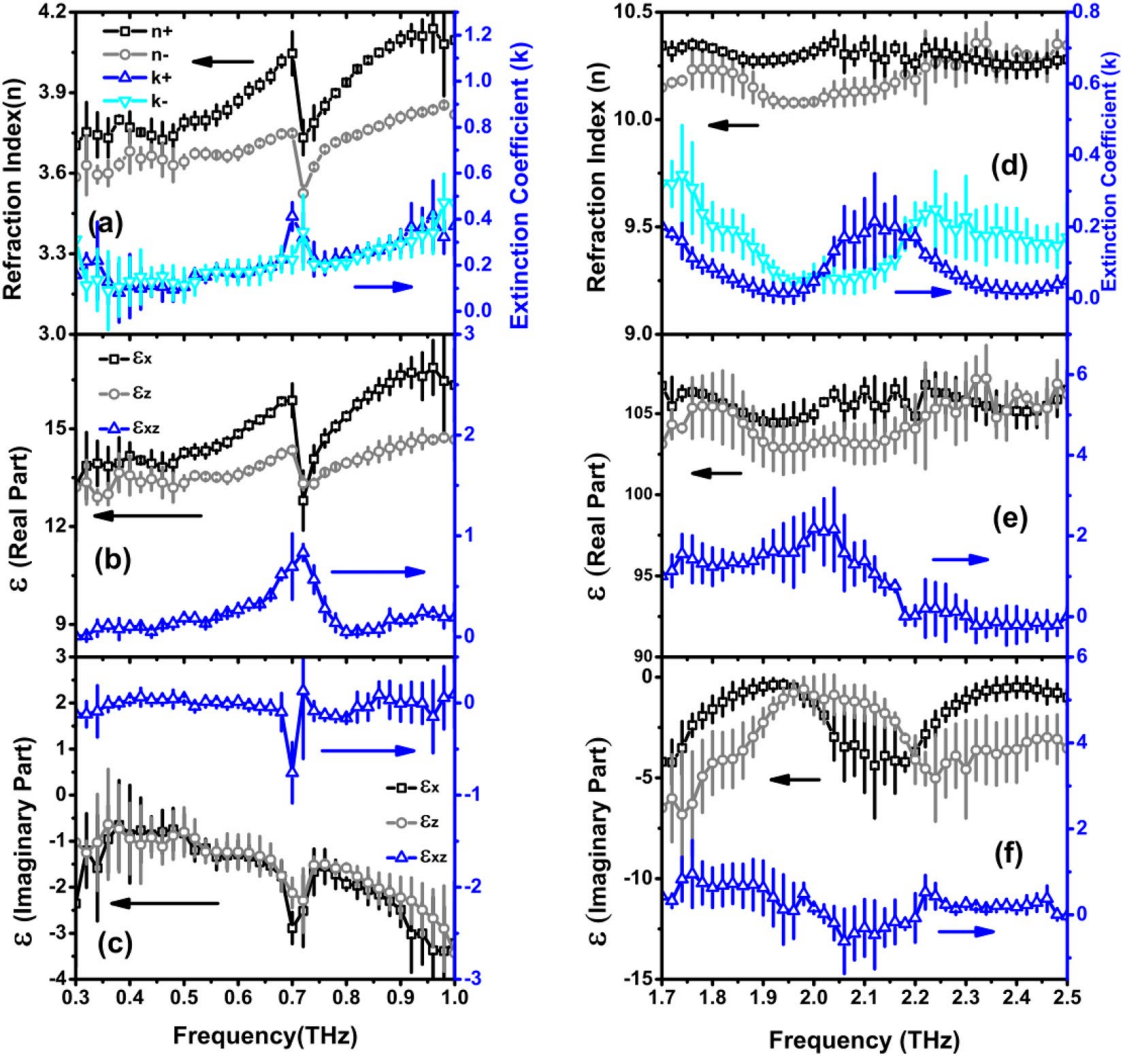
Extracted complex refraction indexes for two eigenmodes and three elements of complex tensor *ε*(*ω*). (**a**) and (**d**) are refraction indexes and extinction coefficients. (**b**) and (**e**) are the real part of *ε*(*ω*), and (**c**), (**f**) show the imaginary part.

The extracted complex refraction indexes for each eigenmode and the three tensor elements of permittivity for the BGSe crystal in THz frequency range are shown in Fig. 4. In our experiments, three BGSe (010) samples with thickness of 0.349, 0.494 and 0.658 mm are used for experimental measurements and data calculations. The error bars associated with the data points indicate the standard deviation. Since there is no transmission signal between 1.0–1.7 THz, and the spectral data below 1.0 THz and those above 1.7 THz show dramatic difference, the low and high-frequency data are presented separately with different scales. A prominent feature is the extremely large refraction index (>10) for both eigenmodes at high frequency (>1.7 THz(, which is very unusual in natural materials. Also, there are broad dips existing in extinction coefficient data in the same frequency range. These features match the observation that the wavepacket-like high-frequency components exist in the time-domain waveforms and propagate at a very low velocity.

From the data of polarization dependent Raman spectroscopy, the phonon resonant peaks at 0.71, 2.08 and 2.38 THz were identified (See Supplementary Fig. S1). In the low-frequency data of Fig. 4, a resonant absorption with Lorentzian lineshape also occurs at 0.71 THz, indicating that the energy unidirectionally flows from EM field to lattice oscillations and finally dissipates in the crystal. This is the normal situation observed in many experiments. On the contrary, there is no such resonant absorption existing in data around 2.08 and 2.38 THz. Due to lacking of centrosymmetry in monoclinic crystals, all Raman active modes are also infrared active. Therefore, there must be other mechanisms responsible for THz responses of BGSe crystal observed above. One probable explanation is the excitation of phonon-polaritons, resulted from the strong coupling between EM field and coherent lattice oscillations. In this case, the EM field and lattice exchange energy with high efficiency, which means the energy exchange rate is much higher than the dissipation and dephasing rates of lattice excitations. As a result, the polariton wave could propagate in the crystal effectively and the lattice’s intrinsic oscillations could be greatly broadened and shifted.

**Figure 5:**
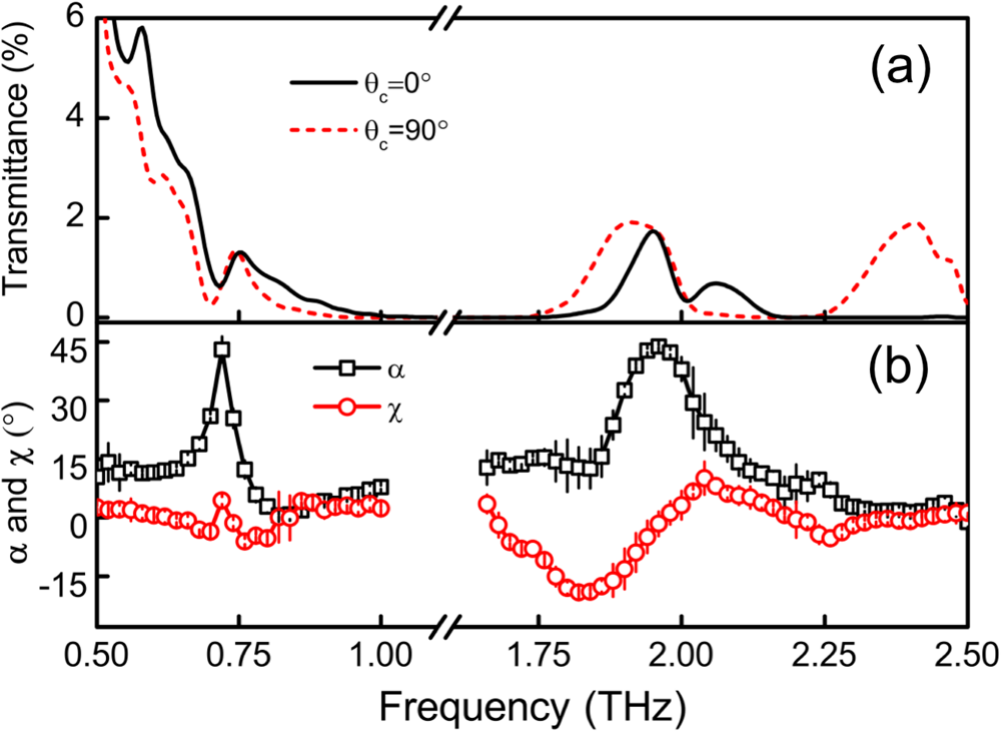
(**a**) Energy transmittance of the BGSe crystal when *θ*_*c*_ is 0° and 90°. (**b**) Extracted major axis direction (*α*) and ellipticity (*χ*) of the eigenmodes.

Figure 5(a) shows the total energy transmittances for two settings of the crystal’s orientation, which were obtained after the summation of energy transmittances of the two orthogonal linearly polarized projection components. There are a much stronger frequency and orientation dependence in the high-frequency spectra, showing the different propagation mechanism. With the obtained matrix elements of permittivity, the major axis direction of ellipse and ellipticity for eigenmodes can be uniquely determined as analyzed before. Figure 5(b) shows the two important factors for one eigenmode because the major axes of the two modes are orthogonal to each other and both have the same chirality and ellipticity. The major axis direction and the ellipticity are heavily dispersive with frequency, especially in the region bearing the phonon resonance. Around 0.71 and 2.0 THz, the major axis almost reaches to 45°. Relatively, the variation of ellipticity is moderate. These results suggest that the full analysis based on eigenmode expansion is necessary to study the property of THz field propagating in monoclinic crystals. The description frame based on usual index ellipsoid analysis is no longer valid for monoclinic crystals when absorption is not negligible.

**Figure 6:**
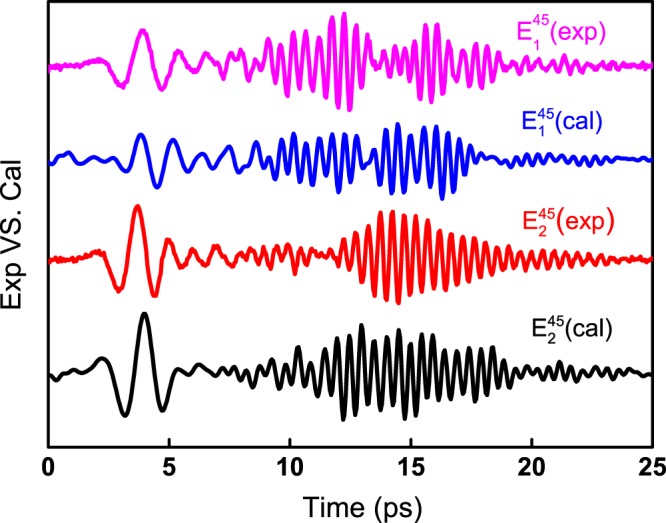
Simulated waveforms for *θ*_*c*_ = 45° using extracted *ε*(*ω*) tensor elements comparing to experiment measured data.

To further confirm our data acquisition and analyzing processes, we used *ε*(*ω*) tensor elements to simulate the transmitted field when *θ*_*c*_ was set to 45°. In this calculation, the measured reference waveform was used as the incident THz field. The simulated and experimentally measured results are shown in Fig. 6. The calculated waveform agrees well with the directly measured data. It is another proof of the effectiveness of our analysis method and the accuracy of the experimental measurements. For BGSe crystal, there still remains a tensor element *ε*_*yy*_ to determine. It is a relatively trivial task once a piece crystal with (100) or (001) cutting is measured.

## Conclusion

We have shown that monoclinic crystals only support elliptically polarized eigen-propagating fields that have well-defined dispersion relations and polarization states when incident field travels along the symmetric axis. The two elliptical eigenmodes have the same ellipticity and chirality, but spatially orthogonal principal axes. In parallel to the linearly polarized eigenmodes in birefringence crystals and circularly polarized ones in chiral media, the elliptically polarized eigenmodes in monoclinic crystals stand as its own class. This property originates from the symmetric property of the complex dielectric tensor of monoclinic crystals, which cannot be diagonalized in general. Therefore, the usual index ellipsoid cannot be well established to describe the propagation of EM waves in the crystal when the dissipation of the propagation field has to be considered. Based on polarization sensitive THz-TDS measurements, the data acquisition, analysis, and extraction procedure are established for direct calculation of dielectric tensor *ε* of monoclinic crystal BaGa_4_Se_7_. The spectra of *ε*_*xx*_, *ε*_*zz*_ and *ε*_*xz*_, as well as the complex refraction index of two eigenmodes, are obtained from 0.3 to 1.0 THz and from 1.7 to 2.5 THz. This method can be applied to any general situation without any preconditions and limitations. The measured THz responses of BaGa_4_Se_7_ near some phonon resonances show a strong coupling between coherent lattice oscillations and THz fields, resulting in the effective excitation and propagation of THz polariton waves in BaGa_4_Se_7_. This phenomenon may lead to development of useful terahertz devices and is worth for further study.

## Electronic supplementary material

Supplementary Information
