# Supplementary material for: Propagation of terahertz waves in a monoclinic crystal BaGa4Se7
Source: Sci Rep. 2018 Nov 1;8:16229. doi: 10.1038/s41598-018-34552-y (PMC6212440; doi:10.1038/s41598-018-34552-y)
Supplement: Supplementary file 1 — Supplementary Information [file 41598_2018_34552_MOESM1_ESM.pdf]

Propagation of terahertz waves in a monoclinic crystal $BaGa_4Se_7$

Yiwen E[1], Jiyong Yao[2], and Li Wang[1,*]

[1]Beijing National Laboratory for Condensed Matter Physics, Institute of Physics, University of Chinese Academy of Sciences, Chinese Academy of Sciences, Beijing 100190, China

[2]Key Laboratory of Functional Crystals and Laser Technology, Technical Institute of Physics and Chemistry, University of Chinese Academy of Sciences, Chinese Academy of Sciences, Beijing 100190, China

[*]**Corresponding Author: wangli@aphy.iphy.ac.cn**

1. EIGENMODES IN MONOCLINIC CRYSTALS

Eigenmodes are a set of special solutions of wave equations, which can maintain the polarization state during propagation. Maxwell's equations are employed to describe the behavior of electromagnetic (EM) waves in a certain crystal:

$$\begin{aligned} \boldsymbol{k} \times \boldsymbol{E} &= \omega \boldsymbol{B} \\ \boldsymbol{k} \times \boldsymbol{H} &= -\omega \boldsymbol{D} \\ \boldsymbol{k} \cdot \boldsymbol{D} &= 0 \\ \boldsymbol{k} \cdot \boldsymbol{B} &= 0 \end{aligned} . \tag{S1}$$

The crystal property is described through the complex permittivity $\varepsilon(\omega)$ based on the constitutive relations:[1]

$$\begin{aligned} \boldsymbol{D} &= \varepsilon_0 \varepsilon(\omega) \boldsymbol{E} \\ \boldsymbol{B} &= \mu_0 \mu(\omega) \boldsymbol{H} \end{aligned} , \tag{S2}$$

where ε_0 and μ_0 are vacuum permittivity and permeability. The wave equation characterizing the propagation is derived from equation (S1) and (S2):

$$\boldsymbol{k} \times \boldsymbol{k} \times \boldsymbol{E} + \omega^2 \varepsilon_0 \mu_0 \varepsilon(\omega) \mu(\omega) \boldsymbol{E} = 0. \tag{S3}$$

In the case of nonmagnetic monoclinic crystal, $\mu(\omega) = 1$ and $\varepsilon(\omega)$ is a complex tensor resulted from the crystal symmetry:

$$\varepsilon(\omega) = \begin{pmatrix} \varepsilon_{xx} & 0 & \varepsilon_{xz} \\ 0 & \varepsilon_{yy} & 0 \\ \varepsilon_{zx} & 0 & \varepsilon_{zz} \end{pmatrix}, \tag{S4}$$

where $\varepsilon_{xz} = \varepsilon_{zx}$. Accordingly, decomposing equation (S3) for a monoclinic crystal in its dielectric frames (x, y, z), we have:

$$\begin{pmatrix} \omega^2\varepsilon_0\mu_0\varepsilon_{xx} - k_y^2 - k_z^2 & k_xk_y & \omega^2\varepsilon_0\mu_0\varepsilon_{xz} + k_xk_z \\ k_yk_x & \omega^2\varepsilon_0\mu_0\varepsilon_{yy} - k_x^2 - k_z^2 & k_yk_z \\ \omega^2\varepsilon_0\mu_0\varepsilon_{zx} - k_zk_x & k_zk_y & \omega^2\varepsilon_0\mu_0\varepsilon_{zz} - k_x^2 - k_y^2 \end{pmatrix} \begin{pmatrix} E_x \\ E_y \\ E_z \end{pmatrix} = 0. \tag{S5}$$

Assume that a plane wave propagates along the y-axis ($k_x = k_z = 0$ and $E_y = 0$)that is the normal of the monoclinic crystal's mirror plane. Equation (S5) is reduced as:

$$\begin{pmatrix} \omega^2\varepsilon_0\mu_0\varepsilon_{xx} - k_y^2 & \omega^2\varepsilon_0\mu_0\varepsilon_{xz} \\ \omega^2\varepsilon_0\mu_0\varepsilon_{xz} & \omega^2\varepsilon_0\mu_0\varepsilon_{zz} - k_y^2 \end{pmatrix} \begin{pmatrix} E_x \\ E_z \end{pmatrix} = 0. \tag{S6}$$

For nontrivial solutions of the electric field, the determination of the matrix in equation (S6) must equal to zero. We obtain the dispersion relations for two eigenmodes that are labeled as "±" respectively:

$$k_y^{\pm} = \frac{\omega}{c}\sqrt{\frac{\varepsilon_{xx} + \varepsilon_{zz} \pm \varepsilon_m}{2}} \equiv k^{\pm}. \tag{S7}$$

Here, $\varepsilon_m = \sqrt{(\varepsilon_{xx} - \varepsilon_{zz})^2 + 4\varepsilon_{xz}^2}$. Substituting equation (S7) into equation (S6), we find that the components E_x and E_z of the eigenmodes satisfy the following relations:

$$\frac{E_z^{\pm}}{E_x^{\pm}} = \frac{-(\varepsilon_{xx} + \varepsilon_{zz} \pm \varepsilon_m)}{2\varepsilon_{xz}} \equiv \sigma_{\pm}. \tag{S8}$$

It is sssumed that the normalized eigen vetor set for eigenmodes is ($\boldsymbol{e}^{\boldsymbol{+}}$, $\boldsymbol{e}^{\boldsymbol{-}}$), which describes the polarization state for each mode. Plane wave $\boldsymbol{E}(y)$ propagating in the monoclinic crystal along the y-axis can be expressed as a superposition of the eigenmodes with the amplitude $E^{\pm}$:

$$\boldsymbol{E}(y) = E^{+}e^{ik^{+}y}\boldsymbol{e}^{\boldsymbol{+}} + E^{-}e^{ik^{-}y}\boldsymbol{e}^{\boldsymbol{-}}. \tag{S9}$$

To express the eigenmode amplitudes $E^{\pm}$ by corresponding components $E_x^{\pm}$ and $E_z^{\pm}$ in dielectric frames, a transformation matrix D is introduced to diagonalize the following equation:

$$\begin{pmatrix} E^{+} & 0 \\ 0 & E^{-} \end{pmatrix} \begin{pmatrix} \boldsymbol{e}^{\boldsymbol{+}} \\ \boldsymbol{e}^{\boldsymbol{-}} \end{pmatrix} = \begin{pmatrix} E_x^{+} & E_z^{+} \\ E_x^{-} & E_z^{-} \end{pmatrix} DD^{-1} \begin{pmatrix} \boldsymbol{x} \\ \boldsymbol{z} \end{pmatrix} = \begin{pmatrix} E_x^{+} & E_z^{+} \\ E_x^{-} & E_z^{-} \end{pmatrix} \begin{pmatrix} \boldsymbol{x} \\ \boldsymbol{z} \end{pmatrix}. \tag{S10}$$

After a straightforward calculation, the transformation D and its inverse matrix D^{-1} are found:

$$D = \begin{pmatrix} \sigma_- & \sigma_+ \\ -1 & -1 \end{pmatrix}, D^{-1} = \frac{1}{\sigma_- - \sigma_+} \begin{pmatrix} 1 & \sigma_+ \\ -1 & -\sigma_- \end{pmatrix}. \tag{S11}$$

Then, we have

$$
\begin{aligned}
\begin{pmatrix} E_x^+ & E_z^+ \\ E_x^- & E_z^- \end{pmatrix} DD^{-1} \begin{pmatrix} \boldsymbol{x} \\ \boldsymbol{z} \end{pmatrix} &= \frac{1}{\sigma_- - \sigma_+} \begin{pmatrix} E_x^+ & E_z^+ \\ E_x^- & E_z^- \end{pmatrix} \begin{pmatrix} \sigma_- & \sigma_+ \\ -1 & -1 \end{pmatrix} \begin{pmatrix} 1 & \sigma_+ \\ -1 & -\sigma_- \end{pmatrix} \begin{pmatrix} \boldsymbol{x} \\ \boldsymbol{z} \end{pmatrix} \\
&= \frac{1}{\sigma_- - \sigma_+} \begin{pmatrix} E_x^+\sigma_- - E_z^+ & E_x^+\sigma_+ - E_z^+ \\ E_x^-\sigma_- - E_z^- & E_x^-\sigma_+ - E_z^- \end{pmatrix} \begin{pmatrix} \boldsymbol{x} + \sigma_+\boldsymbol{z} \\ -\boldsymbol{x} - \sigma_-\boldsymbol{z} \end{pmatrix} \\
&= \frac{1}{\sigma_- - \sigma_+} \begin{pmatrix} E_x^+\sigma_- - E_z^+ & 0 \\ 0 & E_x^-\sigma_+ - E_z^- \end{pmatrix} \begin{pmatrix} \boldsymbol{x} + \sigma_+\boldsymbol{z} \\ -\boldsymbol{x} - \sigma_-\boldsymbol{z} \end{pmatrix}.
\end{aligned} \tag{S12}
$$

The equation (S8) has been used here. Therefore,

$$
\begin{aligned}
E^+ &= E_x^+\sigma_- - E_z^+ = -E_x^+(\sigma_+ - \sigma_-) \\
E^- &= E_x^-\sigma_+ - E_z^- = E_x^-(\sigma_+ - \sigma_-)
\end{aligned} \tag{S13}
$$

and the base vectors for eigenmodes can be expressed as the counterparts in dielectric frames,

$$
\begin{aligned}
\boldsymbol{e}^+ &= C_1(\boldsymbol{x} + \sigma_+\boldsymbol{z}) \\
\boldsymbol{e}^- &= C_2(\boldsymbol{x} + \sigma_-\boldsymbol{z})
\end{aligned} \tag{S14}
$$

C_1 and C_2 are two constants for normalization. Using the relation $\sigma_+\sigma_- = -1$, we have

$$
\begin{aligned}
\boldsymbol{e}^+ &= (\boldsymbol{x} + \sigma_+\boldsymbol{z})/\sqrt{1+A^2} \\
\boldsymbol{e}^- &= (\sigma_+\boldsymbol{x} - \boldsymbol{z})/\sqrt{1+A^2}
\end{aligned} \quad , (\sigma_+ \equiv Ae^{i\phi}) \tag{S15}
$$

It is noticed that $\boldsymbol{e}^+ \cdot \boldsymbol{e}^- = 0$, which means they are not orthogonal in the complex space, but each one is orthonormal with the complex conjugate of the other.

2. POLARIZED ROMAN SPECTROSCOPY

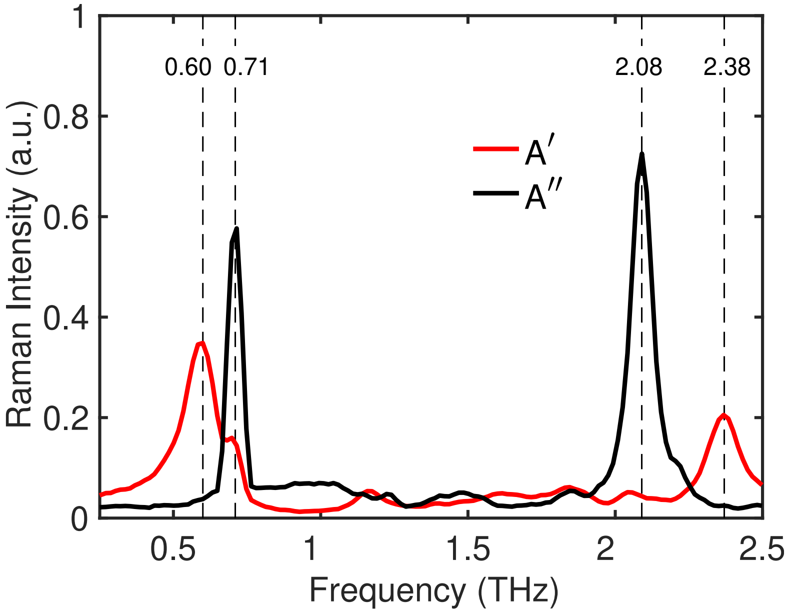

Figure S1. Polarized Raman Spectra. Four modes are observed from 0.3 to 2.5 THz. The modes at 0.71 and 2.08 THz belong to the A′ type Raman tensors. And the other two (0.60 and 2.38 THz) are A″type.

Figure S1 shows the polarized Raman spectra of $BaGa_4Se_7$ (BGSe). The crystal belongs to C_s (m) point group, which has two different Raman tensors (A′ and A″).[2] As shown in the plot, there are four phonon modes. The modes at 0.71 and 2.08 THz are A′ type, which means the Raman signal and the excitation field have the same polarization. On the other hands, the polarization of A″ modes (0.60 and 2.38 THz) are perpendicular to the polarization of the excitation field.

REFERENCES

[1] Born, M. & Wolf, E. *Principles of optics: electromagnetic theory of propagation, interference and diffraction of light* (Elsevier, 2013).

[2] Kuzmany, H. *Solid-state spectroscopy: an introduction* (Springer Science & Business Media, 2009).
